# Mice in Bion-M 1 Space Mission: Training and Selection

**DOI:** 10.1371/journal.pone.0104830

**Published:** 2014-08-18

**Authors:** Alexander Andreev-Andrievskiy, Anfisa Popova, Richard Boyle, Jeffrey Alberts, Boris Shenkman, Olga Vinogradova, Oleg Dolgov, Konstantin Anokhin, Darya Tsvirkun, Pavel Soldatov, Tatyana Nemirovskaya, Eugeniy Ilyin, Vladimir Sychev

**Affiliations:** 1 Institute for Biomedical Problems, Russian Academy of Sciences, Moscow, Russia; 2 Moscow State University, Biology Faculty, Moscow, Russia; 3 Bio-Visualization, Imaging and Simulation Technology Center (BioVIS), NASA Ames Research Center, Moffett Field, California, United States of America; 4 Indiana University, Department of Psychological and Brain Sciences, Bloomington, Indiana, United States of America; 5 Anokhin Institute of Normal Physiology, Russian Academy of Medical Sciences, Moscow, Russia; 6 Kurchatov NBIC-center, National Research Centre “Kurchatov Institute”, Moscow, Russia; Medical University of Graz, Austria

## Abstract

After a 16-year hiatus, Russia has resumed its program of biomedical research in space, with the successful 30-day flight of the Bion-M 1 biosatellite (April 19–May 19, 2013). The principal species for biomedical research in this project was the mouse. This paper presents an overview of the scientific goals, the experimental design and the mouse training/selection program. The aim of mice experiments in the Bion-M 1 project was to elucidate cellular and molecular mechanisms, underlying the adaptation of key physiological systems to long-term exposure in microgravity. The studies with mice combined *in vivo* measurements, both in flight and post-flight (including continuous blood pressure measurement), with extensive *in vitro* studies carried out shortly after return of the mice and in the end of recovery study. Male C57/BL6 mice group housed in space habitats were flown aboard the Bion-M 1 biosatellite, or remained on ground in the control experiment that replicated environmental and housing conditions in the spacecraft. Vivarium control groups were used to account for housing effects and possible seasonal differences. Mice training included the co-adaptation in housing groups and mice adaptation to paste food diet. The measures taken to co-adapt aggressive male mice in housing groups and the peculiarities of “space” paste food are described. The training program for mice designated for *in vivo* studies was broader and included behavioral/functional test battery and continuous behavioral measurements in the home-cage. The results of the preliminary tests were used for the selection of homogenous groups. After the flight, mice were in good condition for biomedical studies and displayed signs of pronounced disadaptation to Earth's gravity. The outcomes of the training program for the mice welfare are discussed. We conclude that our training program was effective and that male mice can be successfully employed in space biomedical research.

## Introduction

After a 16-year hiatus, Russia resumed in 2013 its program of biomedical research in space, with the successful 30-day flight of the Bion-M 1 biosatellite (April 19–May 19, 2013), a specially designed automated spacecraft dedicated to life-science experiments. “M” in the mission's name stands for “modernized”; the epithet was equally applicable to the spacecraft and the research program. The principal animal species for physiological studies in this mission was the mouse (*Mus musculus*). Unlike more recent space experiments that used female mice, males were flown in the Bion-M 1 mission. The challenging task of supporting mice in space for this unmanned, automated, 30-day-long mission, was made even more so by the requirement to house the males in groups.

Russian biomedical research in space traditionally has employed dogs, rats, monkeys, and more recently Mongolian gerbils. The flight of Laika in 1957 was one of the early dog experiments and became world famous for demonstrating that a living organism can withstand rocket launch and weightlessness, thus paving the way for the first human spaceflight. Laika's success also promoted biomedical research with other non-human animals in space that culminated with the Bion biosatellites program. A total of 212 rats and 12 monkeys were launched on 11 satellites and exposed in microgravity for 5.0–22.5 days between 1973 and 1997. Animal experiments on the Bion missions have contributed comprehensive data on adaptive responses of sensorimotor, cardiovascular, muscle, bone and other systems to spaceflight conditions and the mechanisms underlying these adaptations [1], [2].

The use of mice for space experiments offers numerous advantages. Probably the most apparent one is their small size and thus the possibility of utilizing more animals per flight, thus increasing scientific output and the cost-efficiency ratio. Comparisons of data obtained with mice, with those obtained from larger species or humans can also reveal how factors affecting adaptation to spaceflight conditions depend on the size of the organism. The mouse has become the most prevalent “mammalian model” in biomedical research, with a fully described genome and an established role in genetically engineered mutants. While mice are preferred mammalian models for molecular biology studies, their small size is a debated limitation rather than an advantage for physiological studies. Miniaturization of scientific hardware has reduced some of the disadvantages of the species small size. Finally, the use of genetically controlled mice offers a means to reduce inter-individual variability and obtain potentially more consistent results.

Despite the advantages of the mouse as a model organism for space research, their use was rather limited (apart from a number of experiments with mice during early space exploration in the 1950's and 1960's, which were aimed primarily at testing if living organisms can survive the launch or a brief exposure in microgravity) [3]. Flight experiments with mice were performed aboard STS-90 (“NeuroLab”), STS-108, STS-129, STS-131 (“Mouse Immunology I”), STS-133 (“Mouse Immunology II”), and STS-135 with exposure times ranging from 12 to 16 days. Research programs of these flights were largely focused on studies of muscle, bone/tendon/cartilage, nervous, and cardiovascular systems, and innate and acquired immune responses. Experiments were performed with groups of 30 or fewer female C57BL/6J mice, which were dissected typically shortly after return [4]–[9].

The Mice Drawer System (MDS) experiment of the Italian Space Agency is by far the longest spaceflight of mice to date [10]. In this mission, 6 mice were exposed for 91 days aboard the International Space Station. The advantages offered by the possibility of genetic manipulations with mice were utilized in this experiment; three mice were transgenic with pleiotrophin overexpression (C57BLJ10/ PTN) and three mice were their wild-type counterparts. The MDS habitats required periodic replenishment and servicing by by astronauts. Sadly, half of the mice died during the course of this mission due to various estimated reasons.

In the present paper we aim to present a brief overview of the Bion-M 1 mission scientific goals and experimental design. Of particular interest we will focus on the program of mouse training and selection for the experiments, and some outcomes of the Bion-M 1 mission.

### Overview of the Bion-m 1 mission

The scientific program of the Bion-M 1 project was aimed at obtaining data on mechanisms of adaptation of muscle, bone, cardiovascular, sensorimotor and nervous systems to prolonged exposure in microgravity and during post-flight recovery. To this aim, functional measurements *in vivo* were combined with complementary morphological, biochemical, cellular and molecular studies performed *in vitro*. To our knowledge, Bion-M 1 is the first study with mice to follow the dynamics of re-adaptation to normal gravity or to assess post-flight *in vivo* functions.

The *in vivo* measurements in-flight included video recording of mice behavior and continuous recording of arterial pressure during all stages of the flight. After the flight, testing of animal behavior, memory and learning, vestibular function, physical activity, strength and fitness, as well as continuous registration of cardiovascular parameters were performed. The set of *in vivo* measurements was designed to provide data on the effects of microgravity on the physiological systems of interest, and to follow the time course of re-adaptation to Earth's gravity after the flight. To reduce the possibility of inadvertently stressing the mice with numerous post-flight tests and thus obscuring the effects of spaceflight, the decision was made to monitor mice in their home cage using an automated data collection system (TSE PhenoMaster) and implantable telemetry, while reducing the number of tests [11]. The program had to be performed with a limited number of animals; thus the use of a repeated measures design. Mice were tested before and after the flight (or its simulation on-ground) [12].

The *in vitro* studies were focused on metabolism and the structure and function of tissues from microgravity-exposed mice. More specifically, the individual studies were selected to maximize the scientific yield and targeted the impact of microgravity and other factors specific to spaceflight on biochemical, morphological and, primarily, cellular and molecular mechanisms involved in the adaptation of main physiological systems, such as the central nervous system, sensory systems, skeletal muscle, supporting tissues, including bone and connective tissues, cardiovascular, respiratory, digestive, neuroendocrine and immune systems.

The following organs and tissues were harvested: brain structures, vestibular organs, retina and crystalline lens, spinal cord, muscles of hind limbs and back, vertebral column, fore and hind paw bones, articular and cartilage structures, ligaments, bone marrow, cardiac muscle, arteries from brain and hind legs, lung, intestines, liver, kidney, spleen, thymus, skin, salivary glands, testis and blood. These samples were analyzed using morphological (light and electron microscopy, histochemistry and immunohistochemistry), biochemical and a variety of molecular-biology techniques (enzyme-linked immune assays, high-pressure liquid chromatography, western-blot, real-time PCR, whole-genome gene expression). Functional properties of skeletal muscle, cardiac muscle and resistive arteries samples were evaluated using myography and atomic force microscopy. Mice for *in vitro* studies were euthanized by cervical dislocation; because of the diversity and number of studies, this method was chosen as the common procedure to reduce variability and one that provided the least interference with the objectives of the *in vitro* studies.

## Materials and Methods

### Ethical statement

The study was approved by IACUC of MSU Institute of Mitoengineering (Protocol № 35, 1 November, 2012) and of Biomedical Ethics Commission of IBMP (protocol № 319, 4 April, 2013) and conducted in compliance with the European Convention for the Protection of Vertebrate Animals used for Experimental and Other Scientific Purposes [13].

### Animals

Experiments were performed with C57BL/6N male mice. The choice of strain was based on the requirements of the tissue harvesting program participants and the fact that C57/BL6 is one of the most widely used strains. The selection of male mice was based on the same requirement. The advantages offered by using males are their substantially larger size and the absence of sex steroid cycling compared to females; the vast majority of animal research is conducted using males, so their use in this study made the results more readily comparable to the data in the published literature. Nevertheless, the risks associated with group-housing mature males were recognized and greatly affected the preparatory procedures. It was decided that the optimal age of mice at launch was 4–5 months, when the males were still young but fully mature. The growth rate in mice is known to slow down by this age, and obesity, which C57/BL6 mice are prone to exhibit, is not yet considered a factor.

Space environment affects microorganisms by increasing their virulence; major shifts in immune status in space are well documented [5], [8]. It is reasonable to think that even pseudo pathogenic species may increase morbidity during prolonged exposure in space [14], [15]. These considerations underscored the necessity for the use of specific pathogen-free mice in the experiments, a side benefit of this approach being the reduced variability of data obtained in SPF-animals [16].

Male C57BL/6N mice (n = 300) weighing 22–25 g were purchased from the Animal Breeding Facility - Branch of Shemyakin & Ovchinnikov Institute of Bioorganic Chemistry. Mice were specific pathogen free. The main site for mouse training was the animal facility of Moscow State University's Institute of Mitoengineering. At the time of launch and the start of the related control experiments, the mice were 19–20 weeks old.

Mice were individually identified using RFID chips (El1000, Felixcan, Spain) and earmarks. Routine manipulations of the mice consisted of daily handling, examination, and weighing.

### Experimental groups

The Bion-M 1 biosatellite, launched on April 19, 2013 from cosmodrome Baikonur, and the descent module landed on May 19, 2013 in the vicinity of Orenburg, successfully fulfilling the plan for an unmanned, 30-day-long orbital spaceflight. Housing and climate parameters were replicated in the subsequent ground control (GC) experiment (July 26 to August 26, 2013). A total of 4 experimental groups were used for the flight and ground control experiments (n = 45 per group). An additional fifth group included the backup mice for the main flight group (n = 45). Mice of the space flight group (SF) were exposed to microgravity for 30 days. Concurrent with the SF mice, another group of 45 mice remained in the animal facility (SFV). The ground control (GC) experiment was conducted, after the landing, in the refurbished BOS flight habitats. The habitats were installed in a climatic chamber that replicated the temperature, humidity, gas composition and other flight-specific climate parameters. The corresponding vivarium control (GCV) mice were housed in the animal facility. The separate and concurrent GCV groups were used to account for possible seasonal differences between SF and GC mice.

Each of the groups (SF, GC, SFV, GCV and backup SF mice) included mice designated for *in vivo* studies and recovery (n = 10) and mice for dissection and *in vitro* measurements (n = 35). Each *in vivo* study subgroup, in its turn, consisted of 5 mice implanted with telemetry probes to monitor blood pressure and 5 intact animals.

**Figure 1:**
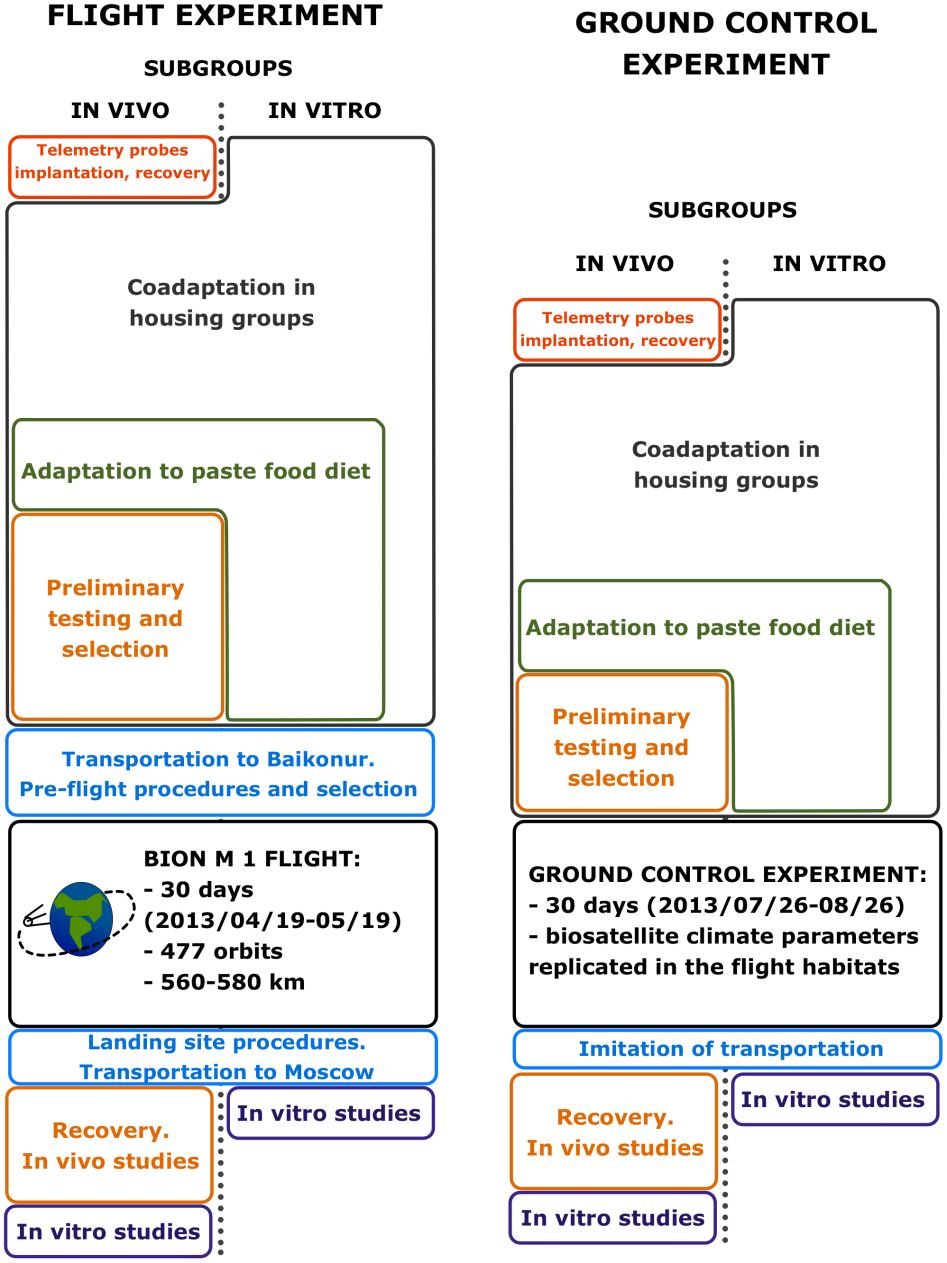
Schematic diagram of the experiments. Mice of SF, SFV and backup SF groups were co-adapted in housing groups of 3 mice each, SF mice were adapted to paste food diet, and mice of *in vivo* subgroups passed through preliminary tests. After transportation to and adaptation at Baikonur, SF mice were flown aboard the Bion-M 1 satellite for 30 days. After landing mice were examined and transported to Moscow, where animals of the *in vitro* subgroup were dissected, while recovery dynamics was followed in the *in-vivo* subgroup before dissection 7 days after landing. Ground control experiment replicated the principal stages of the spaceflight experiment.

Mice were handled and trained before the flight and ground control experiments. Basically, training consisted of shaping the groups of three mice each for social housing and adaptation to paste diet. The training of mice designated for *in vivo* studies was more comprehensive. It started with implanting the telemetry probes and, following recovery, a set of preliminary behavioral and functional tests (Figure 1, Table 1 and 2).

**Table 1:** Key dates of the flight experiment.

FLIGHT EXPERIMENT: key dates	Time relative to Launch (L) or Return (R)
*Arrival of mice from the breeding facility*	L-51d
*RFID-chips implantation, ear marking*	L-50d
*Telemetry probes implantation*	L-50d
*Weighing, examination*	L-50d - L-3d
*Co-adaptation of mice in groups of three*	L-45d - L-3d
*Adaptation of mice to paste diet*	L-14d - L-3d
*Training/tests with mice designated for in vivo studies*	L-30d - L -9d
*Transportation to Baikonur*	L-7d
*Adaptation at launch site*	L-7d - L-3d
*Final selection of the flight group*	L -3d
*Transfer of mice to the habitats*	L-3d
*Installation of habitat assemblies into the biosatellite*	L-3d
*Monitoring of mice in habitats*	L-3d - L-1d
*Transportation of the biosatellite for further assembling*	L-2d
*Transportation to launch site*	L-1d
*Installation of the rocket at launch table*	L-16h
*Launch – 2013/04/19 14:00*	L 0
*Bion-M 1 flight – 477 orbits – 30 days (2013/04/19-05/19)*	
*Braking engine start*	R -0.75h
*Atmospheric entry*	R-0.25h
*Parachute opening*	R-0.17h
*Touchdown engines firing (2013/05/19 07:11)*	R 0
*Recovery team arrival*	R +0.25h
*Dismounting of habitat assemblies*	R+2h-R+3h
*Mice examination at recovery site*	R+2h – R+5h
*Transportation to Moscow*	R+5h-R+12h
*Euthanasia and in vitro studies*	R+13h – R+25h
*In vivo tests and recovery monitoring*	R+0d – R+7d
*Euthanasia of the recovery group, in vitro studies*	R+7d

**Table 2:** Key dates of the on-ground control experiment.

CONTROL EXPERIMENT: key dates	Time relative to experiment Start (S) or End (E)
*Arrival of mice from the breeding facility*	S-38d
*RFID-chips implantation, ear marking*	S-37d
*Telemetry probes implantation*	S-36d
*Weighing, examination*	S-37d – S-2d
*Co-adaptation of mice in groups of three*	S-29d – S-2d
*Adaptation of mice to paste diet*	S-14d – S-2d
*Training/tests with mice designated for in vivo studies*	S-21d – S-6d
*Transportation to IBMP*	S-1d
*Transfer of mice to the habitats*	S-1d
*Installation of habitat assemblies into the climate chamber*	S-1d
*Monitoring of mice in habitats*	S-1d-S0
*Control experiment start (2013/07/26 14:00)*	S0
*Ground control experiment – 30 days (2013/07/26-08/26)*	
*Control experiment end (2013/08/26 09:00)*	E0
*Dismounting of habitat assemblies*	E+1h – E+2h
*Mice examination*	E+1h – E+3h
*Transportation imitation*	E+4h – E+8h
*Euthanasia and in vitro studies*	E+8h – E+23h
*In vivo tests and recovery monitoring*	E+0d – E+7d
*Euthanasia of the recovery group, in vitro studies*	E+7d

### Housing

The BOS habitats were designed to house up to three mice per group [17]. Stable cohorts of three mice were maintained throughout the training program and consequent experiments, with the exception of brief periods of post-surgery recovery (5–6 days) and behavioral tests (7 days), when they were housed individually.

Individually ventilated cages (GM 500, Techniplast, floor area 500 cm^2^) were used. Techniplast mice shelters (red plastic triangles, 15Lx11Wx8H cm), paper tubes and paper tissues for nesting material were provided to enrich the environment. Cage cleaning was done once weekly, and water was refreshed twice weekly. All materials were sterilized prior to use by autoclave. Techniques aimed to prevent animal contamination were thoroughly followed.

### Habitats

**Figure 2:**
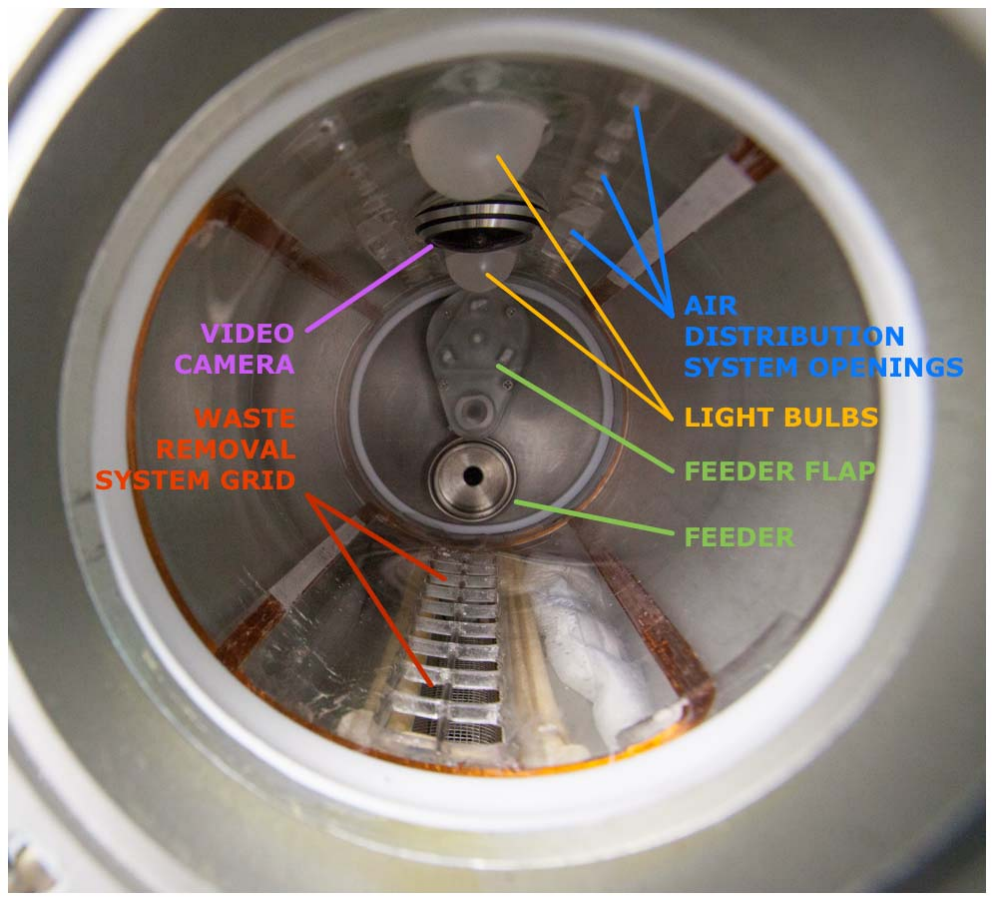
Habitat interior.

The hardware for mice housing in-flight was an adapted version of the habitats used for rats in the original Bion program. It was developed by Biofizpribor, according to the IBMP requirements and was tested by IBMP in 2011. Each block consisted of five individual habitats (BOS, Block Obespecheniya Soderzhaniya, Russian for - literally - Unit for the Provision of Housing). The living compartment of each habitat was an acrylic cylinder 98±2 mm in diameter and 200±10 mm in length with a total volume of approximately 1.7 l (Figure 2). A stainless steel feeder (9 ml) was mounted at one end (the “top”) of the cylinder. The feeder had a flap, used in the refill procedure. The food distribution system (except for the feeders) was common to all five habitats in the block. Paste “space” food was pumped from the container and distributed between individual habitats of the assembly 6 times daily (every 4 hours at 00:00, 04:00, 08:00, 12:00, 16:00 and 20:00), a total of 54 g/day per 3 mice. Food distribution was adjusted through manipulations of pressure developed in the container and in the feeders to follow this pattern prior to experiments.

Bulbs for day-phase (08:00–20:00, 45±5 Lux) and night-phase (20:00–08:00, 5±2 Lux) lighting, an air supply fan (0.3 m/s) and an air distribution system were positioned on one side of the cylinder. A video camera was installed between the light bulbs; video was recorded 6 times daily (simultaneously with feeding, each session lasted 2 hours) at 8 frames per second. The opposite side of the cylinder had the grid for waste removal. The waste removal system was individual for each habitat. The door at the “bottom” of the cylinder had a glass window. In order to minimize impact of variability arising from possible differences in mice housing conditions, experimental subgroups were formed from mice that were housed in different habitats.

Air composition and climate parameters in the satellite were maintained by the centralized life support system and remained in the designed range throughout the flight.

### Diet

During the initial stage of training, mice were provided with standard pelleted food (Assortiment Agro, Russia) and deionized water given *ad libitum*. Two weeks prior to launch, and the start of the GC experiment, the animals were adapted to the flight paste food diet. Both the paste and standard diets (pelleted food and water) were first presented together; after 2–3 days the water bottle was removed, followed in another 2–3 days by the removal of the pelleted food. Vivarium control (SFV and GCV) groups were fed standard chow and water throughout the experiments. The “space” paste food diet was developed at IBMP by T. Gurieva and E. Mednikova. It was produced based on the standard chow, with water and casein as gelling agent added.

Consumption of standard pelleted chow and water (in SFV and GCV) or paste diet (in SF and GC) was monitored daily for ten days in ten randomly chosen cages per group. Data for the cage was averaged across the days and expressed as g per 10 g bodyweight.

A separate preliminary experiment in metabolic chambers was conducted to quantify urination in mice fed standard chow and paste food. Four groups of mice, of 3 mice each, were housed in metabolic chambers (Techniplast, Italy) and fed pelleted food (grinded) or paste food. Body weight, food and water consumption, urine volume and feces weight were monitored daily (3 days for each diet).

### Telemetry probes implantation

Arterial pressure was monitored in sub-groups of mice designated for recovery, using implantable probes and telemetry hardware (Data Science International, USA). The hardware was adapted and integrated to the Bion-M biosatellite by engineers from Biofizpribor (St. Petersburg, Russia) and the Centre national d'études spatiales (CNES, France). Miniature probes (model PA-C10) weighing 1.4 g were implanted 3 weeks before the start of experiments, following the manufacturer's instructions. The catheter of the probe was implanted into the left carotid so that its tip just reached the aorta [18]. The body of the transmitter was implanted subcutaneously on the flank, briefly described below.

Mice were anaesthetized by an intraperitoneal injection of a solution containing tiletamine/zolazepam (15 mg/kg each) combined with xylazine (3 mg/kg). Depth of anesthesia was assessed regularly by hind leg pinch and observation of respiration rate, and maintained by additional dose(s) of the mixture (about 20% of the initial dose) as needed. A midline incision was made on the ventral neck. The carotid artery was separated and ligated 1–2 mm cranially to its bifurcation. A second ligature was used to suspend the artery 4–5 mm proximal to the site of ligation. Through a small incision in the artery wall, the catheter was advanced 6–7 mm towards the aorta and fixed in place with sutures and acrylic glue. A subcutaneous pocket on the flank of the animal was made with blunt scissors, and the transmitter was inserted there with a small hemostat. The catheter and the transmitter were fixed in place with acrylic glue and the skin incision closed with absorbable sutures. During the recovery period, mice were housed individually; they were under daily veterinarian control and received ibuprofen 4 mg/ml and Bactrim 4 mg/ml in drinking water.

### Pre-flight training of mice designated for in vivo studies

Mice for the *in vivo* studies were trained and tested before the experiments to select homogenous groups and to benefit from repeated measures in experimental design; the latter consideration was particularly important in light of relatively small number of animals.

On the morning of day 0 mice a battery of behavioral and functional tests was administered. First, animals were examined for visible abnormalities in exterior and simple reflexes [19]. *The Open field test* was performed in a round, 60 cm arena of black plastic with a grey floor and 40 cm high walls [20]. Mouse behavior was video recorded at 25 fps for 10 minutes under a bright white light (200 Lux) followed by 10 min in darkness (IR light). Videos were analyzed for locomotor activity and other behavioral parameters using EthoVision v. 8.5 (Noldus). *Grip force* was measured using Grass FT-03 force transducer [21]. Analog output was amplified, digitized at 1 kHz sampling rate and analyzed using PowerGraph software (InteropticaS, Russia). *RotaRod* test was conducted using UgoBasil hardware with rotation speed increased from 6 to 50 rpm in 3 min, 5 trials were performed with each mouse [22].

On the evening of the same day, animals were placed into cages of *Phenomaster* (TSE Instruments, Germany), an automated data collection system, where they were monitored for 7 days. The system was equipped with an infrared grid to register animal activity, scales for the water bottle and feeder, a running wheel with unrestricted access and rotation counter, an “operant wall” for positively reinforced operant conditioning [23]. There were 16 such cages, housing mice individually. Measures were read once per minute using TSE software. Blood pressure and heart rate in mice, instrumented with telemetry probes were continuously recorded throughout the tests.

*Operant conditioning* sessions were conducted automatically, once a day and started at 22:30, controlled by IntelliMaze software control. The first session was designed to reinforce nosepokes emitted by an animal while both light stimuli (left and right) were active. This session was used to establish individual baseline tendencies to display nosepokes to the right or left. On subsequent days, mice were trained to direct nosepokes to the side opposite to their initial bias. The number of rewarded responses was limited to 50; each sweetened dry milk pellet weighed 14 mg. To promote learning, mice were maintained at 90% ad lib body weight. Access to food was provided throughout the day from 07:30 until 19:00–20:00. Reward pellets were the alternative source of food.

The quality of blood pressure signal (for the operated mice), probabilities of running wheel use, combined with performance in conditioning task were used as criteria for inclusion into experimental groups. Selected mice were randomly assigned to either flight or ground control or vivarium groups.

### Transportation

The groups of mice designated for flight were transported to Cosmodrome Baikonur one week before launch. Transportation was by car and by air in several steps and lasted approximately 24 h. Containers of nontransparent blue plastic 40×60 cm with 4 sub-compartments (each for a group of three mice) equipped with air filters (Animal Breeding Facility - Branch of Shemyakin & Ovchinnikov Institute of Bioorganic Chemistry) were used. Paste food was provided to mice during transportation.

### Statistical analysis

One-way ANOVA (transportation bodyweight data) followed by Bonferroni's multiple comparisons was performed with transportation bodyweight data. In order the pre/post flight bodyweight data set met the assumptions of normality and homoscedasticity intrinsic to the ANOVA model, rank transformation was applied to the entire set of observations [24] prior to two-way ANOVA followed by Bonferroni post-hoc comparisons; noteworthy, the results of the test on raw and transformed data were identical. Fisher's exact test (survival), Man-Whitney U-test (home cage food and water consumption, open field test behavioral data) or paired Student's t-test (metabolic chambers data) was performed using GraphPad Prism software (version 6.00 for MacOS, GraphPad Software, USA).

## Results and Discussion

### Group housing

Living conditions for animals considered optimal on earth cannot be provided in the confines of a space satellite. Microgravity, apart from purely technical limitations of the housing hardware, may disrupt normal mouse behavior, and an inability to express natural behavior is posited as a potential cause of distress in laboratory animals [25]. This and more obvious negative factors, such as accumulated waste, suboptimal feeding regimen etc., can negatively affect mice and could increase aggressiveness in space habitats. As a result we considered aggressive interactions a major risk factor. To minimize this risk, we specifically sought to shape the groups for social housing and carefully select stable groups prior to experiments.

From an initial set of 300 male mice, 88 groups of 3 mice were formed, a total of 53 groups were used for the space flight (SF) experiment and corresponding SFV, 35 groups for subsequent GC and GCV studies (87%). During the co-adaptation period, a total of 12 groups had to be rearranged (14%).

Male mice are known to have aggressive tendencies. Their aggressiveness depends on a number of factors, including strain (C57/BL6 is considered one of the more aggressive strains), living space and other housing conditions and, in the case of group housing, the history of group interactions is a major factor [17]. In an animal facility, aggressive interactions are easily overcome by simply removing the aggressor from a group or by individual housing of mature males. Individual housing, however, precludes all social interactions and thus compromises the animals' overall welfare. For this reason, environmental enrichment is often employed in animal facilities, with the goal of reducing aggressive interactions.

While the analysis of measures promoting co-habitation of male mice was beyond the scope of the current project, several pertinent observations were made. We reasoned that environmental enrichment and daily handling were critically important for successful co-adaptation of mice and the development of sustainable hierarchy in the group. In a preliminary experiment, when no environmental enrichment was used, only 50% of mice (out of a sample of 42) successfully co-adapted to living in groups of three. The enrichment used to prepare mice for the Bion-M 1 experiments (shelters, nesting material and paper tubes) proved to be useful in two ways. Shelter with good qualities is salient to mice, and male mice will compete for the resource [26]. Additionally, adequate shelter facilitates the observation of nesting behavior within the group. Nesting behavior can differentiate groups, with stable groups building a communal nest within a chamber, whereas mice that cannot co-adapt as a group compete and fight for the shelter, with one or two mice building a separate nest outside of the chamber.

Handling and other simple manipulations are known to induce an acute stress reaction in mice, seen as an increase in blood pressure, heart rate, body temperature, and plasma corticosterone [27]–. At the same time, handling is often used to habituate mice and rats to experimental manipulations (mild stress), with different handling techniques having different impacts on their behavioral indices [31]. In the course of training for the experiments, mice were handled daily to monitor signs of fighting and body weight, and to help identify the provokers and recipients of aggression. Frequent handling proved critically important to condition and calm mice to some of the tests. For instance, the “air righting” test used to evaluate vestibular function involved holding and rotating individual mice cupped in the palms of the experimenter's hands prior to releasing the mouse into a safe fall. Extensive handling and homecage enrichments yielded male mice that were tractable and cooperative during such potentially provocative procedures. Other, useful and informative observations could be made after a change of bedding. Mice become extremely active when placed in a fresh cage. We have repeatedly observed that in unstable groups mice would fight promptly after a change of bedding. In summary, fighting episodes observed after handling or cage cleaning, along with an absence of a communal nest, were treated as indication that the group had to be either rearranged or split and excluded from the experiments.

### Food and water consumption

The average pelleted food consumption in SFV and GCV groups was 1.30±0.14 g/10 g bodyweight (BW) and their water consumption was 1.52±0.65 g/10 g BW (m±sd). Average paste food consumption in the Space Flight and the Ground Control groups was 5.52±0.88 g/10 g BW. Taking into account the 76–78%, water content of paste food, dry weight consumed was the same as that for pelleted food of 1.27±0.20 g/10 g BW (p = 0.4514, Mann Whitney test). Notably, water intake via the paste food diet was 4.25±0.67 g/10 g BW, or roughly three times *ad libitum* water consumption, when water was presented separately from dry, pelleted diet (p<0.0001, Mann Whitney test). Apparently the need for nutrients, rather than thirst, governed the paste food consumption.

**Figure 3:**
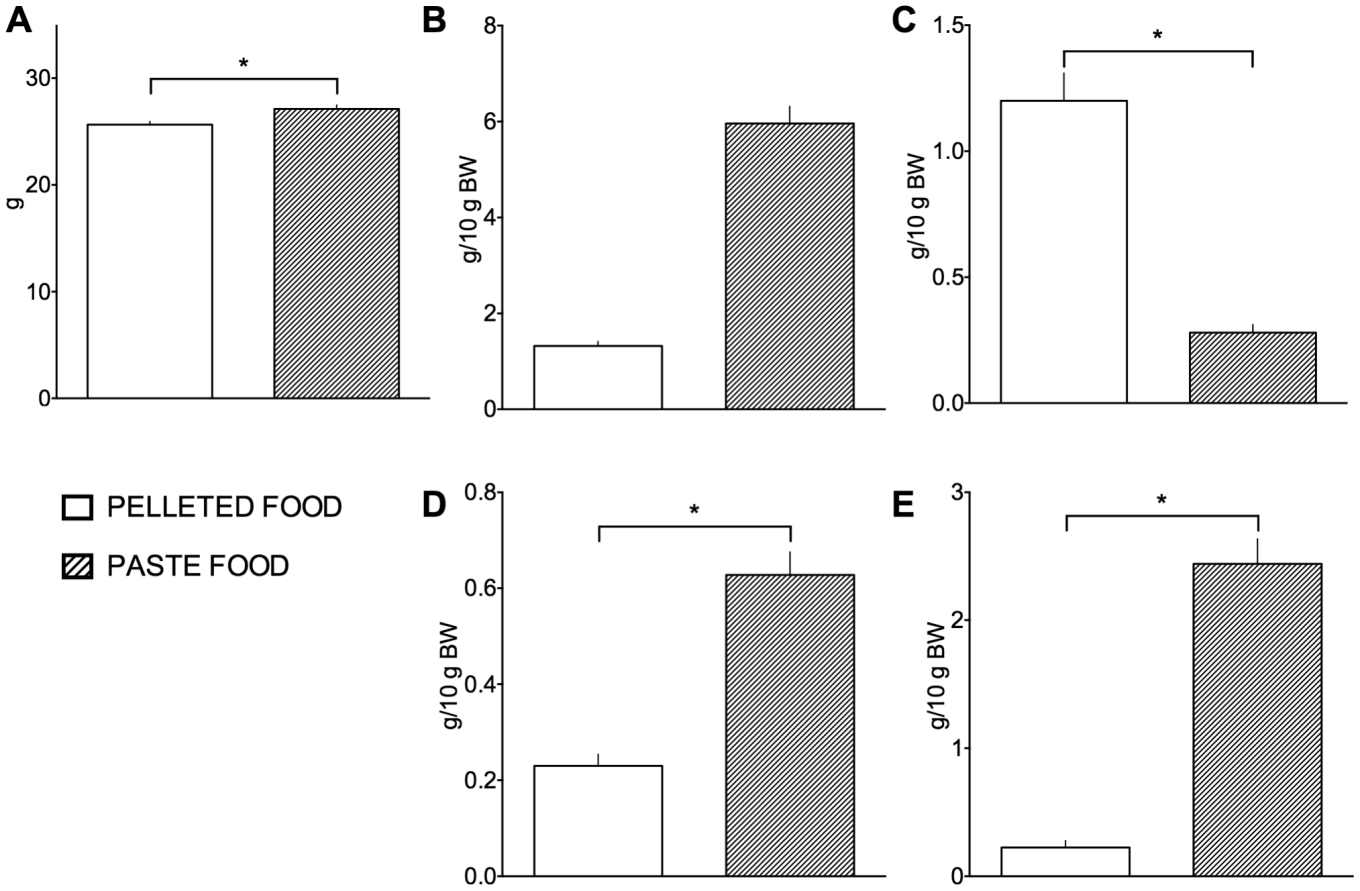
Metabolic parameters in mice fed pelleted food and paste diet. Body weight (A), food (B) and water (C) consumption, feces (D) and urine (E) production. Differences significant at p<0.05 are marked with an asterisk. When the paste food diet with high water content was introduced mice displayed an increase in bodyweight, stopped drinking, while diuresis and feces weight were increased indicating excretion of excess water consumed with the paste diet.

While no adverse impact of this increased water intake was evident, mice maintained on paste diet produced more urine that was more dilute than normal. When paste food was introduced, water consumption decreased roughly 5 times its original amount; body weight increased by 1.5±0.4 g, and feces weight tripled (Figure 3). Measured urine production in mice fed pasted food diet was 2.4 ml/10 g BW, which was 10 times the value in mice fed standard diet (Student's paired t = 12.55, df = 3, p = 0.0011).

### Telemetry probes impact on the mice welfare

Continuous blood pressure measurement, with implantable telemetry probes, was an integral part of the *in vivo* studies. The health status and functional state of the implanted mice were carefully monitored after surgery because the novelty of this procedure as part of a long spaceflight constituted a major risk factor for the mission.

**Figure 4:**
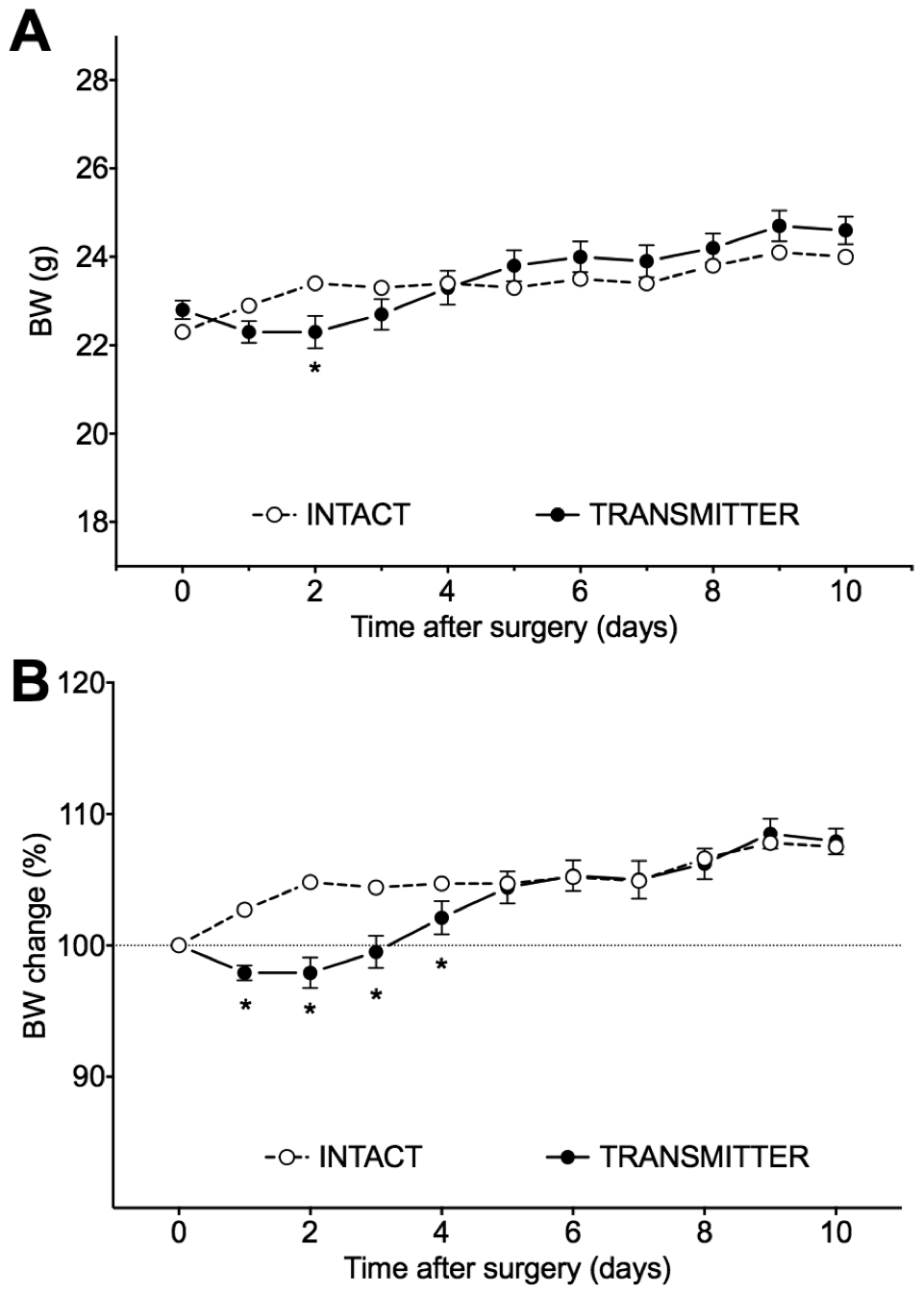
Bodyweight (A) and relative BW change (B) after telemetry probe implantation. Differences significant at p<0.05 are marked with an asterisk. As can be considered from bodyweight data, acute recovery was over by day 5 after surgery.

Thirty-five (35) mice were implanted with PA-C10 blood pressure transmitters. The mice tolerated with apparent ease telemetry probe implantation. As can be seen from body weight data, the acute recovery was complete by day 5 post-surgery (Figure 4). At approximately the same time, mice were behaviorally recovered in terms of locomotor activity and nest building, and were reunited with their co-habitants.

Tests of corneal, pineal and other simple reflexes, performance on RotaRod, grip strength, running wheel performance, and learning and memory tests revealed no adverse effects of probe implantation. We occasionally noted, slight eyelid drooping. Although this ptosis could be interpreted as indicating compromised brain blood supply, we do not believe this was the case. Indeed, the circle of Willis is incompletely developed in mice, with 10% incidence in the C57 strain [32]; the right common carotid artery was ligated during the surgery. The absence of impaired motor or cognitive functions in implanted mice contradicts interpretations of brain blood supply deficiency. An extracranial blood supply through the external carotid could, however, be compromised in operated mice, particularly in the most distal branches of the artery that supply blood to the eyelid. We surmise that was the reason for the slight ptosis observed on the side ipsilateral to the carotid ligation.

Mice that underwent surgery were housed individually during acute recovery period (5 days on average) and were re-introduced to their co-habitants when recovered. Two modes of reunion were tested. One method was to introduce two un-operated mice to the cage of an operated animal. This was designed to exploit “host-intruder” paradigm and offer an advantage to the host (operated  =  ”weak” mouse) due to concerns for its safety. In nearly all cases, however, the non-operated “intruders” were the ones to suffer, when the implanted mouse assumed the dominant position. The second method was to introduce all three mice to a fresh cage; this was found to be safe for both operated and intact mice. Based on *in vivo* tests conducted before the experiment, we selected the latter mode of reunion. To summarize, when partially recovered from the operation, mice with implanted probes can be easily group-housed with intact mice and display little (if any) differences from mice without surgery.

### Pre-flight training of mice designated for in vivo studies

Preliminary training and tests were performed with 42 mice for both the flight (SF) experiment and the corresponding vivarium control (SFV), and with 30 mice for the ground control (GC and GCV groups) experiment (Table 3); 26 (62%) and 16 (53%) mice correspondingly fulfilled the inclusion criteria. Here, it is important to mention that probabilities of fulfilling each of the criteria are below 1. The probabilities of mice displaying a good BP signal, running vigorously in the wheel, performing well in discriminative learning, and maintaining amicable interactions with cagemates should be treated as independent and therefore should be multiplied to get the joint probability. Although the rule is common knowledge, it is easy to overlook when planning an experiment, and anticipating sufficient allowance of extra animals.

**Table 3:** The outcome of the pre-flight procedures with mice for in vivo studies.

Criterion	Flight + Flight vivarium			Ground control + Ground control vivarium		
	fulfilled, n	failed, n	fulfilled, %	fulfilled, n	failed, n	fulfilled, %
*Blood pressure signal quality*	15	5	75	12	3	80
*Voluntary running more than 3 km/day*	34	8	81	25	5	83
*More than 95% correct reactions in the operant conditioning task by day 3*	39	3	93	28	2	93
*Housing group stability (cages)*	19	2	90	14	1	93

### Transportation

**Figure 5:**
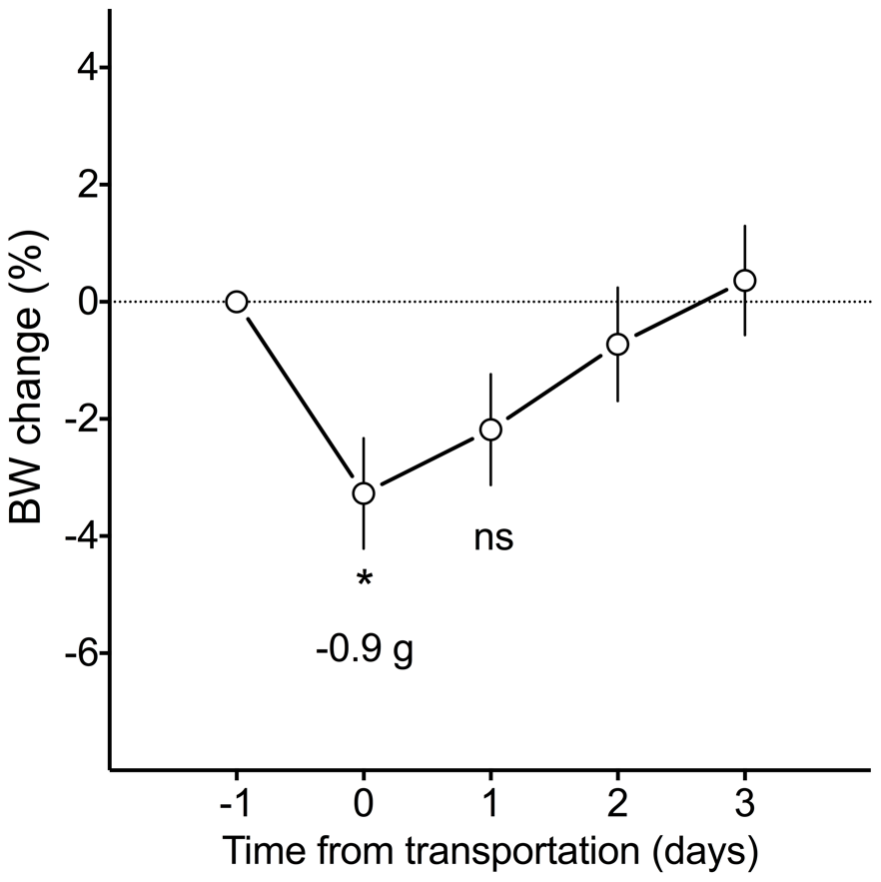
Mice bodyweight dynamics after transportation to the launch site. Differences significant at p<0.05 are marked with an asterisk. Transportation did not seriously affect the mice, as can be concluded from a slight drop of bodyweight and its rapid recovery.

The flight group of mice was transported to Cosmodrome Baikonur one week before launch. The relatively long (24 h) transportation to Baikonur did not seriously affect the mice as evidenced by a slight bodyweight change of −3% (−0.9 g, Figure 5). Bodyweight returned back to pre-transportation values in two days.

## General Outcomes

### Climate

The environmental parameters in the biosatellite during the orbital mission, in the climatic chamber during the ground control experiment, and in the animal facility for the VC groups (Table 4), were closely matched and within the acceptable range for the housing of rodents [33], [34]. The climate parameters aboard the satellite were within the designed range throughout the flight [35].

**Table 4:** Climate parameters in flight, control experiments and the animal facility.

Parameter		Animal facility	Biosatellite	Control experiment
*Temperature, °C*	*m±sd*	23.0±1.5	21.1±0.4	21.3±0.8
	*min-max*	21.0–27.0	19.5–22.9	20.3–22.3
*Relative humidity, %*	*m±sd*	49±17	61±3	71±2
	*min-max*	20–75	53–65	68–73
*Ambient pressure, mmHg*	*m±sd*	na	762±6	747±3
	*min-max*	na	752–770	742–753
*O_2_, %*	*m±sd*	na	21.2±1.1	21.2±0.5
	*min-max*	na	19.3–21.2	20.6–21.2
*CO_2_, %*	*m±sd*	na	0.002±0.005	0.0682±0.0718
	min-max	na	0.000–0.015	0.0201–0.2096

na – data not available.

### Mice condition after the flight and control experiments

In the flight (SF) experiment 16 out of 45 mice (36%) survived and in the ground control (GC) experiment, 38 out of 45 mice (84%) survived. It should be noted that loss of all mice (n = 15) in one assembly during space flight was a result of a gross malfunction of the food distribution system. Taking into account that the flight was aimed at exposing mice in the microgravity at, rather than evaluating the long established habitat system, survivorship would be calculated as 16 out of 30 mice (53%). Loss of mice in the SF group was obviously higher than in GC (p<0.0001, Fisher's exact test); however, the results of the control experiment are in concordance with data obtained during preliminary evaluation of the system, where none of the 15 mice died during a 30-day housing in the habitats (p = 0.1760, Fisher's exact test). Though the comparison of mice survival in our 30-day automated mission with the data obtained in the 91-day MDS experiment aboard the ISS, where astronauts attended to the mice and survival rate was 50% (3 out of 6), might be dubious, numerically the values do coincide. Upon examination of the surviving SF mice, 4 (25%) had limb injuries and 6 (38%) had various tail injuries. These results were quite unexpected taking into account that, during the preliminary evaluation of the system, all the mice remained in perfect health after 30 days of housing in the habitats.

### Mice behavior during the flight

To better understand and interpret the fatalities and injuries sustained by some of the mice in the two functioning habitat assemblies, we made use of the uniquely rich video data that were recorded from the Bion habitats (see description, above). It should be noted that never before have such extensive video records been collected and that this design feature alone could earn the “modernization” tag in the Bion-M1 designation.

The library of video recordings from the two habitat assemblies consisted of 2,476, 30-min segments of individual habitats, captured during both light and dark phases of each day of the mission, as described earlier. As might be expected, video quality was superior in the early phases of the flight and visibility was compromised over time, as floating liquids and debris tended to obscure the camera lens and the general atmosphere of the habitats. More specifically, at the beginning of first week of space flight we categorized as clear with maximal visibility 76% of the video segments. There was a precipitous decrease in visibility at the beginning of the ensuing weeks: the proportion of such clear segments was only 3% and 6% for the 2^nd^ and 3^rd^ weeks, respectively. On the first day of the final week none (0%) of the habitats were clearly visible in the video records.

Two fatalities occurred in different habitats on the 2^nd^ day of the flight. In another cage, one mouse died on the 9^th^ day. There was no evidence of fighting, biting, or agonistic behavior in any of the video samples, so we do not believe that any of deaths or injuries resulted from aggression among the males, evidencing success of our effort to train and select stable housing groups. The first two losses occurred early in the flight, so lack of access to food cannot account for them; and again, in no habitat was there visual evidence of competition that prevented access to food.

**Figure 6:**
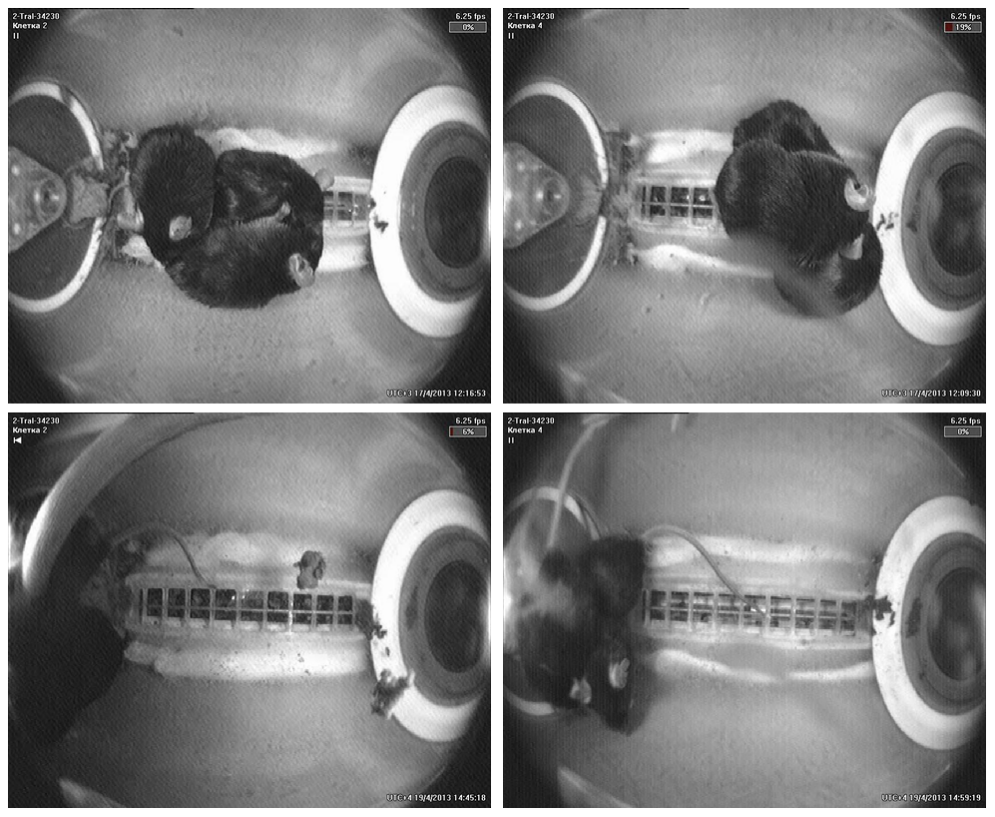
A representative photograph of mice in the flight habitats. Note that mice occupy the floor grid before launch (upper row) and cling to the feeder (lower row) in microgravity. The same cages are shown.

What might help explain the deaths? The video records clearly showed that the mice spent much time in physical contact (huddling) with one another and, significantly we think, they were grouped against the flat, feeder wall of the cylindrical habitat (Figure 6). We quantified their location within each habitat on select days during the flight; choosing days on which the video segments provided good available visibility. Because we were interested in the aggregative behavior of the mice in different areas, we encoded when there were three, two or one individuals in contact with the feeder wall, the blank wall, or were in the middle area of the habitat. When one mouse was in contact with a flat wall (feeder or blank) and one or more mice were in contact with it but not necessarily with the wall itself, we scored such aggregation as being in wall contact. Using these criteria we found that during the 1^st^ day of flight, two or three mice were aggregated at the feeder wall for 80% (median value; range: 63%–100%) of the observable segments. They spent 0% of the time at the blank wall. On the 3^rd^ flight day, aggregations of two or three mice were at the feeder wall for 50% (median; range: 5%–95%) of the observation periods and at the blank wall for a median of 0% (range: 0%–11%). On Day 11, feeder wall aggregations with two or more mice occurred for a median of 46% of the observation time and there were no multiple animal aggregations at the blank wall.

For comparison, we applied the same criteria to the Ground Control habitats for the same days during the simulated flight. Among the GC habitats on day 1, aggregations of two or three mice were seen for a median of 61% of the time (range: 46%–75%) and such aggregations were at the blank wall for only 1.5% of the time (range 0%–4%). On the third and 11^th^ days the median time aggregations of two or three mice were at the feeder wall was 79% and 95.5%, respectively; there were no such aggregations at the blank wall.

In all, these video samples documented from the first day of experimentation, both in microgravity and on earth, that male mice aggregated non-randomly at the feeder wall of the cylindrical habitat. Individuals were often seen oriented toward the food area, which was recessed 1 cm. Whether the behavioral orientation reflected active feeding of the paste diet, or thigmotactic responses to the recess, or grasping of an edge created by the recess or presented by the rotating flap, or some combination of such factors was not discernible from the videos. It was clear, however, that both under 1-g and microgravity conditions, the animals spent most of their aggregative time, which was considerable, near or in contact with the feeder wall. In microgravity, the animals assumed a greater range of orientations at the wall and may have more actively inserted their bodies into the recesses or crevasses created by the hardware, especially the moving parts. This remains supposition, but appears likely and should be considered in future designs as well as in the arrangements for video surveillance during future flights.

Results obtained in the Bion-M 1 mission should be taken into account in design of new mice habitats for future space missions. Despite the general design has long since proven its efficiency in the previous Bion flights with rats, including use of paste food and the system of its distribution, further development is needed to better adapt it for mice based on this study; obviously any moving parts in direct access of the animals should be excluded. The advantage of the habitats design is its ability to support mice without any human interference in relatively long duration missions. This degree of autonomy has not been reached by any other of the existing animal habitats, for instance, in the recent MDS mission the water tank had to be refilled every 10 days, and while food supply was designed to operate automatically for 60 days, eventually the food bars had to be manually advanced as needed by the astronauts [10].

### Examination at the landing site

Examination of mice after the Bion-M 1 flight directly at the landing site (return +3 h) revealed gross motor function impairment: the mice could not maintain steady posture and their fore and hind paws were positioned more to their sides, rather than directly under their trunk (14 of 16, 88%); the mice did not move even when prodded; when lifted, the mice spread their toes excessively (16/16, 100%) and righting reaction was absent or impaired in an aerial righting test (6 of 6, 100%). Some recovery of locomotor function was evident by about 6–8 h after landing, when the mice could keep normal posture and even rear. All mice were found to be exophthalmic. Excessive urinations with almost colorless urine were noted in 14 out of 16 mice.

### Body weight data

**Figure 7:**
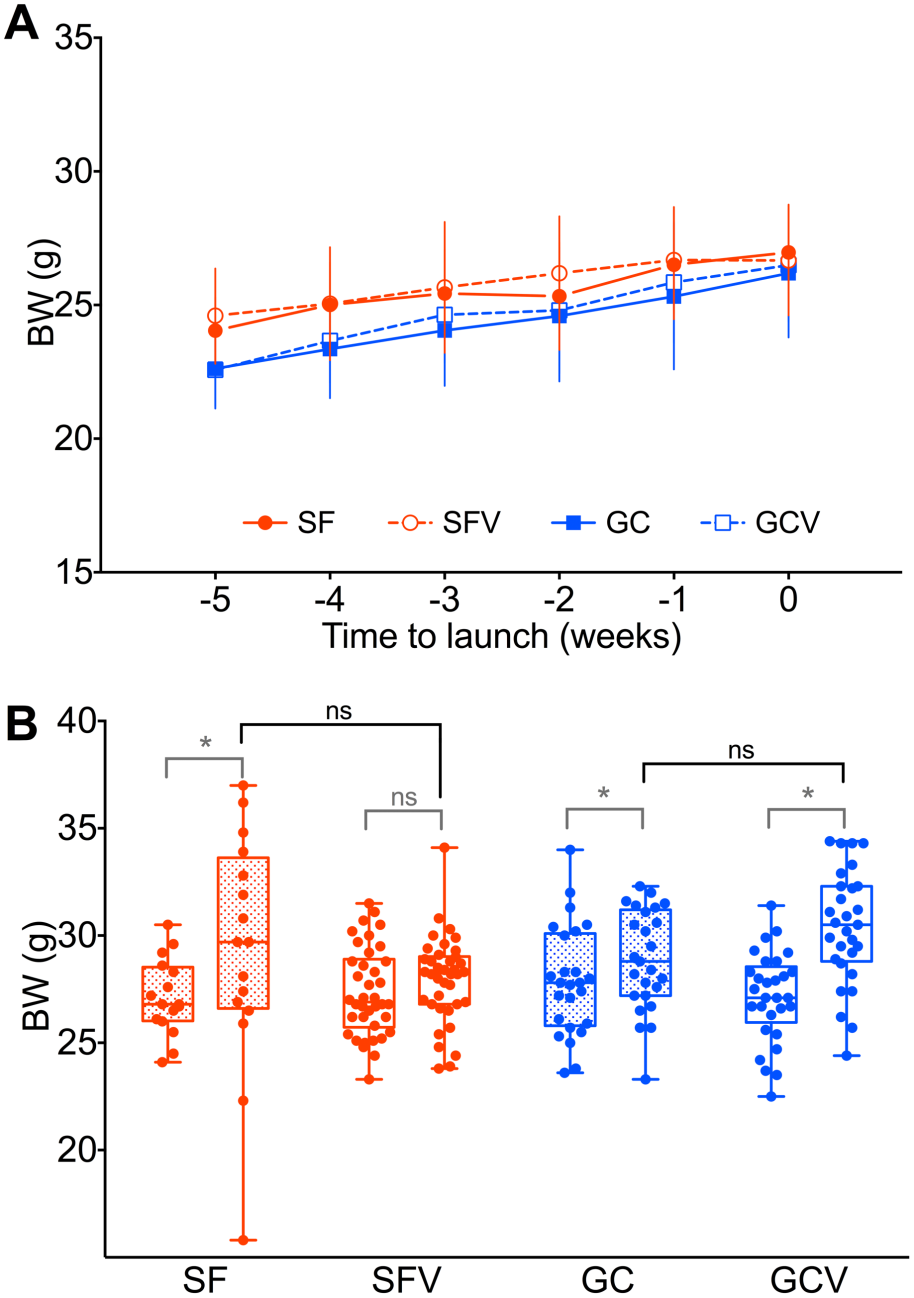
Mice body weight during training (A) and bodyweight before and after the experiments (B). The pairs of bars on the (B) panel represent body weight before (left box) and after (right box) the corresponding experiment. Points represent individual mice data, boxes – lower to upper quartile, whiskers – minimum and maximum. Differences significant at p<0.05 are marked with an asterisk.

The nutritional status of mice post-flight was variable. Whereas the majority of mice either gained some weight or maintained their pre-flight values (12/16, 75%), 1 mouse was famished (BW 50% below pre-flight value), and 3 were distinctly overweight by 30–40%. The differences in bodyweight between spaceflight, ground control and vivarium control groups were not significant (F (3,104) = 1.40, p = 0.2468) and they accounted for only 3% of total variation (Figure 7). Post-hoc comparisons revealed no differences between the groups before the experiments. The comparison of pre- versus post-flight values revealed that bodyweight increased in the flight group (8%, p = 0.0010), ground control (4%, p = 0.0456) and control vivarium groups (11%, p<0.0001) with overall effect of “time” being highly significant (F(1,104) = 59.79, p<0.0001). The relations of main experimental groups (flight and control experiment groups) to the corresponding vivarium controls were mirrored: while the flight group had gained, on average, more than the mice housed in the animal facility, mice from the control experiment group gained less weight than their vivarium control counterparts. We consider that this discrepancy is explained by little opportunity for exercise in the relatively small habitats in microgravity, and partial loss of mice during the flight experiment, leading to excesses of food available to the survivors. In the control experiment, where the effort was made to reproduce the feeding regimen observed in the flight experiment, given that more mice survived, the relative amount of food per mouse was smaller leading to smaller weight gain. Seasonal variations in growth cannot be ruled out either.

### Behavior in the open-field test

**Figure 8:**
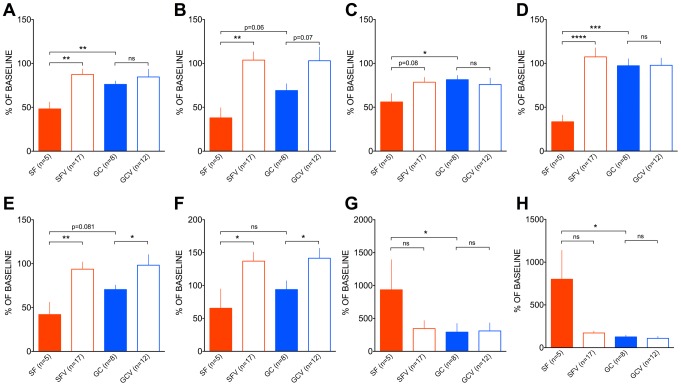
Post-flight open field behavior parameters expressed as percent of pre-flight background values. Total (A), center (B) and periphery (C) distance moved, rearing frequency (D), center entries frequency (E), time in center (F), latency to the first center zone entry (G) and grooming duration (H). SF mice displayed reduced activity compared to any of the control groups (GC, SFV or GCV). Mice housed in habitats (SF and GC) were reluctant to explore the center of the arena. Statistical analysis was performed using Mann-Whitney test (*−p<0.05, **−p<0.01, ***− p<0.005 and ****−p<0.0001, ns – not significant or the p value is indicated).

Behavioral activity of individual mice in the open field was recorded during the training period prior to the start of the experiments and one day after landing (SF and SFV), and at corresponding times for the control groups (GC and GCV); the software for behavioral analyses extracted the nine parameters in Table 5. After return from 30-day spaceflight mice were markedly less active, with both distance (Figure 8A, C) and rearing frequency (Figure 8D) drastically reduced compared to either GC or SFV animals, whereas grooming duration (Figure 8H) was much higher in SF mice compared to any of the control groups. Interestingly, both SF and GC mice who were housed in relatively small habitats displayed more pronounced thigmotaxis compared to mice kept in standard cages (SFV or GCV correspondingly), as evidenced by a decrease of center zone entries (Figure 8E), time spent in center (Figure 8F) and distance moved in center zone, while latency to the first entry into the center zone was increased in SF mice only (Figure 8G). Behavior parameters of mice from two vivarium groups, SFV and GCV, were indistinguishable.

**Table 5:** Open field test.

Parameter		SF (n = 5)	SFV (n = 17)	GC (n = 9)	GCV (n = 12)
*Distance moved, m*	*Pre*	83.9±6.2	84.2±6.3	82.8±4.5	85.7±5.9
	*Post*	39.0±5.5	69.7±3.4	63.2±4.6	69.1±4.3
*Distance moved in center zone, m*	*Pre*	31.2±3.8	34.1±3.2	32.2±3.5	30.4±2.8
	*Post*	10.4±2.1	31.9±1.7	22.1±2.9	29.0±3.1
*Distance moved in the periphery zone, m*	*Pre*	52.7±5.2	50.1±3.7	50.6±2.0	55.3±3.7
	*Post*	28.6±5.1	37.8±2.5	41.1±2.8	40.1±2.4
*Movement speed, m/min*	*Pre*	5.50±0.56	5.01±0.22	4.85±0.26	5.27±0.36
	*Post*	3.45±0.49	4.21±0.23	3.84±0.23	4.23±0.21
*Rearing frequency*	*Pre*	170±14	169±12	147±10	132±11
	*Post*	53±11	166±8	138±7	128±14
*Center zone entry frequency*	*Pre*	83±10	91±8	81±8	80±6
	*Post*	30±6	78±5	56±5	75±6
*Time in center zone, s*	*Pre*	279±50	367±42	367±22	310±23
	*Post*	161±55	434±30	338±46	423±40
*Latency to first center zone entry, s*	*Pre*	13±6	11±2	28±10	20±5
	*Post*	52±13	21±4	37±11	27±9
*Grooming duration, s*	*Pre*	83±31	88±14	72±6	89±6
	*Post*	319±52	114±13	88±14	103±27

The drastic decrease in activity by SF mice in this test is obviously related to adverse impact of prolonged 30-days spaceflight on the sensorimotor systems of these animals. In part, housing within the restricted confines of the habitats decreased the propensity of the mice to enter and explore the center of the arena; such observations are usually interpreted in terms of increased anxiety in these animals compared to cage-housed controls [20]. Compared the flight habitat interior, animals maintained in the animal facility experienced enriched environment and such differences are associated with greater exploration of the central area in the OFT [36]. In contrast, increased housing density, experienced by SF and GC mice, had little effect on the central area exploration. In the present case the higher animal density, sometimes associated with increased central exploration and reduced anxiety [37], did not prevail.

The example of OFT data illustrates the efficacy of our training program and experimental design for promoting scientific output of the studies performed with mice after landing in the Bion-M 1 program. Because OFT activity is known to decrease with repetitive tests, the repeated testing approach used in this study could potentially obscure the effects of spaceflight. Nevertheless, the opposite was true. While some of the measures taken before the flight, e.g., distance moved, were very close in different experimental groups with variation coefficients ranging between 16 and 30%, others differed between the groups and were quite variable within the group, e.g. latency to enter central zone with within group coefficient of variation of 70–110% (Table 5). In order to reduce this inter-individual and seasonal variability, results of preliminary testing were used to make the correction for baseline differences; this transformation made possible differentiation of spaceflight and habitat effects (Figure 8).

### Post-flight adjustment of experimental groups

Due to the partial loss of mice during the flight, the number of animals in other groups had to be adjusted (Table 6). The number of control mice (GC, SFV, GCV) for *in vitro* studies was decreased proportionately to the number of SF animals available. This decision was made basing on the relatively small variability of the measures to be taken in inbred C57 mice [12] and time considerations (complete dissection of one animal lasted 1.0–1.2 hours and dissection of all the mice took more than 24 hours). Taking that accidental death of mice in flight was a random event and that bodyweight data from all the animals was available, the mice for control groups were selected to match the initial bodyweight of SF mice. Different tactics were chosen for the *in vivo* subgroups. The behavioral data were characterized by high variability, at the same time all the animals designated for *in vivo* studies underwent preliminary testing and selection, taking this into account the number of mice for *in vivo* studies was not altered in order to obtain a better estimate of the behavioral parameters in the batch of mice used and to increase the power of the planned analyses.

**Table 6:** Number of animals in experimental groups after post-flight adjustment.

Group	In vitro studies	In vivo studies
*Flight experiment (SF)*	11	5
*Flight vivarium control (SFV)*	18	20
*Ground control experiment (GC)*	16	9
*Ground control experiment – vivarium control (GCV)*	16	13

### Implementation of the scientific program

Despite significant loss of animals in flight, the scientific program was not abridged and was implemented in full. Hemodynamic and behavioral data were collected throughout the flight. Dissection of SF and SFV mice started 12 hours after landing on May 20^th^, 2013. Recovery was followed for 7 days in the *in vivo* subgroup of SF mice and the dissection of these animals was performed on May 26. Joint effort of teams from Russia, USA, France, Germany, Italy and Ukraine who took part in the *in vivo* studies and tissue-sharing program resulted in over 70 distinct hypothesis-driven studies.

## Conclusions

The Bion-M 1 flight was unique in many ways, particularly the unprecedented duration of an automated mission with living animals. The sex of the mice was male, thus imposing greater attention to creation and selection of stable groups of mice for social housing. The overall success was evident: aggressive interactions were noted on single occasions and under conditions of starvation only. The use of male animals is obviously required for research in male reproduction and their larger size compared to females offered clear advantages. Our program of mice training for flight and control experiments of the Bion-M 1 project was aimed to: ensure that mice adapt to stressful conditions of space flight when housed in groups; to collect baseline data from these mice; select homogenous groups; and, more generally, to promote collection of less variable data. The results of our program presented here demonstrate that group housed male mice need not be excluded *a priori*, but quite on the contrary, can be successfully employed in space biomedical research if clear and simple procedures provided by this study are followed.

The failures in this project were connected with hardware malfunction in food distribution system and to microgravity-related changes in mouse behavior. Some of the injuries and deaths encountered in this pioneering effort suggest moving parts within the habitat present potential risks and should be avoided or carefully analyzed in the design of future mouse habitats.

In this space experiment we have, for the first time, introduced implantable telemetry in these small laboratory animals. This shift to data collection during the flight became possible with the development of miniature implantable probes, thereby dramatically increasing the scientific output of such experiments. Moreover, the use of an automated data collection system during the post-flight stage of this experiment provided comprehensive data on the course and mechanism of recovery of varied physiological systems following exposure to microgravity. The unprecedented inclusion of daily video recordings proved valuable for scientific purposes, for animal welfare considerations, and for assessing both nominal and aberrant events.

The good condition of spaceflight mice was the basis for the positive results of both the recovery and tissue sharing studies; leading us to conclude that our training program was generally effective. We express our hope that the results and considerations presented here will be useful for the planning of future missions and will help to ensure the welfare of rodents in space research.

## References

[1] Grigoriev AI, Iliyn EA (2007) Animals in space. Vestnik Rossijskoj Akademii Nauk 77: 963–973.

[2] Ilyin EA (2000) Historical overview of the Bion project. J Gravit Physiol 7: S1–S8. 11543436

[3] Burgess C, Dubbs C (2007) Animals in Space. From Research Rockets to the Space Shuttle. UK: Springer Praxis. 406 p.

[4] Buckey JC, Homick JL, editors (2003) The Neurolab Spacelab mission: neuroscience research in space: results from the STS-90, Neurolab Spacelab mission. Houston: National Aeronautics and Space Administration, Lyndon B. Johnson Space Center. 333 p.

[5] Pecaut MJ, Nelson GA, Peters LL, Kostenuik PJ, Bateman TA, et al (2003) Genetic models in applied physiology: selected contribution: effects of spaceflight on immunity in the C57BL/6 mouse. I. Immune population distributions. J Appl Physiol 94: 2085–2094. 12514166 10.1152/japplphysiol.01052.2002

[6] Behnke BJ, Stabley JN, McCullough DJ, Davis RT, Dominguez JM, et al (2013) Effects of spaceflight and ground recovery on mesenteric artery and vein constrictor properties in mice. FASEB J 27: 399–409. 23099650 10.1096/fj.12-218503 PMC4050424

[7] Gridley DS, Mao XW, Stodieck LS, Ferguson VL, Bateman TA, et al (2013) Changes in Mouse Thymus and Spleen after Return from the STS-135 Mission in Space. PLoS ONE 8: e75097. 24069384 10.1371/journal.pone.0075097 PMC3777930

[8] Baqai FP, Gridley DS, Slater JM, Luo-Owen X, Stodieck LS, et al (2009) Effects of spaceflight on innate immune function and antioxidant gene expression. J Appl Physiol 106: 1935–1942. 19342437 10.1152/japplphysiol.91361.2008 PMC2692779

[9] Lujan HL, Janbaih H, Feng HZ, Jin JP, DiCarlo SE (2006) Reduced susceptibility to ventricular tachyarrhythmias in rats selectively bred for high aerobic capacity. AJP: Heart and Circulatory Physiology 291: H2933–H2941. 16891405 10.1152/ajpheart.00514.2006

[10] Cancedda R, Liu Y, Ruggiu A, Tavella S, Biticchi R, et al (2012) The Mice Drawer System (MDS) Experiment and the Space Endurance Record-Breaking Mice. PLoS ONE 7: e32243. 22666312 10.1371/journal.pone.0032243 PMC3362598

[11] Andreev-Andrievskiy AA, Popova AS, Borovik AS, Dolgov ON, Tsvirkun DV, et al (2014) Physiology & Behavior. 132: 1–9. 10.1016/j.physbeh.2014.03.033 24802359

[12] Festing MF, Overend P, Gaines Das R, Cortina-Borja M, Berdoy M (2002) The design of animal experiments: reducing the use of animals in research through better experimental design. London: Royal Society of Medicine. 120 p.

[13] European Convention for the Protection of Vertebrate Animals Used for Experimental and Other Scientific Purposes: Convention Européenne Sur la Protection Des Animaux Vertébrés Utilisés À Des Fins Expérimentales Ou À D'autres Fins Scientifiques:[Strasbourg, 18. III. 1986]

[14] Aviles H, Belay T, Fountain K, Vance M, Sonnenfeld G (2003) Increased susceptibility to Pseudomonas aeruginosa infection under hindlimb-unloading conditions. J Appl Physiol 95: 73–80. 12626488 10.1152/japplphysiol.00968.2002

[15] Wilson JW, Ott CM, Quick L, Davis R, zu Bentrup KH, et al (2008) Media Ion Composition Controls Regulatory and Virulence Response of Salmonella in Spaceflight. PLoS ONE 3: e3923. 19079590 10.1371/journal.pone.0003923 PMC2592540

[16] Howard BR (2002) Control of variability. ILAR J 43: 194–201. 12391394 10.1093/ilar.43.4.194

[17] Van Loo PLP, Van Zutphen LFM, Baumans V (2003) Male management: coping with aggression problems in male laboratory mice. Laboratory Animals 37: 300–313. 14599305 10.1258/002367703322389870

[18] Kurtz TW (2005) Recommendations for Blood Pressure Measurement in Humans and Experimental Animals: Part 2: Blood Pressure Measurement in Experimental Animals: A Statement for Professionals From the Subcommittee of Professional and Public Education of the American Heart Association Council on High Blood Pressure Research. 10.1161/01.HYP.0000150857.39919.cb 15611363

[19] Schneider I, Tirsch WS, Faus-Keβler T, Becker L, Kling E, et al (2006) Systematic, standardized and comprehensive neurological phenotyping of inbred mice strains in the German Mouse Clinic. Journal of Neuroscience Methods 157: 82–90. 16720049 10.1016/j.jneumeth.2006.04.002

[20] Gould TD, Dao DT, Kovacsics CE (2009) The Open Field Test. In: T.D. Gould (ed.), Mood and Anxiety Related Phenotypes in Mice, Neuromethods. 42. NewYork: Humana press. pp. 1–20.

[21] Maurissen JPJ, Marable BR, Andrus AK, Stebbins KE (2003) Factors affecting grip strength testing. Neurotoxicology and Teratology 25: 543–553. 12972067 10.1016/s0892-0362(03)00073-4

[22] Rogers D, Peters J, Martin J, Ball S, Nicholson S, et al (2001) SHIRPA, a protocol for behavioral assessment: validation for longitudinal study of neurological dysfunction in mice. Neurosci Lett 306: 89–92. 11403965 10.1016/s0304-3940(01)01885-7

[23] Malkki HAI (2010) Appetitive operant conditioning in mice: heritability and dissociability of training stages. Front Behav Neurosci. 4. Article 171. 10.3389/fnbeh.2010.00171 PMC2990458 21119771

[24] Conover WJ, Iman RL (1981) Rank Transformations as a Bridge Between Parametric and Nonparametric Statistics. The American Statistician 35: 124–129.

[25] Newberry RC (1995) Environmental enrichment: increasing the biological relevance of captive environments. Applied Animal Behaviour Science 44: 229–243.

[26] Van de Weerd HA, Van Loo P, Van Zutphen L, Koolhaas JM, Baumans V (1998) Strength of preference for nesting material as environmental enrichment for laboratory mice. Applied Animal Behaviour Science 55: 369–382.

[27] Bouwknecht JA, Hijzen TH, van der Gugten J, Maes RA, Olivier B (2000) Stress-induced hyperthermia in mice: effects of flesinoxan on heart rate and body temperature. Eur J Pharmacol 400: 59–66. 10913585 10.1016/s0014-2999(00)00387-3

[28] van Bogaert MJV, Groenink L, Oosting RS, Westphal KGC, van der Gugten J, et al (2006) Mouse strain differences in autonomic responses to stress. Genes Brain Behav 5: 139–149. 16507005 10.1111/j.1601-183X.2005.00143.x

[29] Drude S, Geiβler A, Olfe J, Starke A, Domanska G, et al (2011) Side effects of control treatment can conceal experimental data when studying stress responses to injection and psychological stress in mice. Lab Animal 40: 119–128. 21427691 10.1038/laban0411-119

[30] Gariépy J-L, Rodriguiz RM, Jones BC (2002) Handling, genetic and housing effects on the mouse stress system, dopamine function, and behavior. Pharmacol Biochem Behav 73: 7–17. 12076720 10.1016/s0091-3057(02)00789-x

[31] Hurst JL, West RS (2010) Taming anxiety in laboratory mice. Nat Meth 7: 825–826. 10.1038/nmeth.1500 20835246

[32] Ward R, Collins RL, Tanguay G, Miceli D (1990) A quantitative study of cerebrovascular variation in inbred mice. J Anat 173: 87–95. 2074233 PMC1256083

[33] National Research Council (2010) Guide for the Care and Use of Laboratory Animals. Washington: National Academies Press. p 41–103.

[34] FELASA (2007) Euroguide On the Accommodation and Care of Animals Used for Experimental and Other Scientific Purposes. London: Royal Society of Medicine. 65 pp.

[35] Available: http://biosputnik.imbp.ru. Accessed 19 December 2013.

[36] Lin E-JD, Choi E, Liu X, Martin A, During MJ (2011) Environmental enrichment exerts sex-specific effects on emotionality in C57BL/6J mice. Behavioural Brain Research 216: 349–357. 20732356 10.1016/j.bbr.2010.08.019 PMC3727905

[37] Morgan JL, Svenson KL, Lake JP, Zhang W, Stearns TM, et al (2014) Effects of Housing Density in Five Inbred Strains of Mice. PLoS ONE 9: e90012. 24658028 10.1371/journal.pone.0090012 PMC3962340

